# Local control and survival after stereotactic body radiation therapy of early-stage lung cancer patients in Slovenia

**DOI:** 10.2478/raon-2023-0032

**Published:** 2023-07-26

**Authors:** Karmen Stanic, Jasna But-Hadzic, Jan Zagar, Martina Vrankar

**Affiliations:** Department of Radiation Oncology, Institute of Oncology Ljubljana, Slovenia; Faculty of Medicine, University of Ljubljana, Ljubljana, Slovenia

**Keywords:** stereotactic body radiotherapy (SBRT), early-stage lung cancer, lung cancer, local control, survival

## Abstract

**Background:**

Stereotactic body radiation therapy (SBRT) precisely and non-invasively delivers ablative radiation dose to tumors in early-stage lung cancer patients who are not candidates for surgery or refuse it. The aim of research was to evaluate local control, overall survival (OS), local progression free survival (LPFS), distant metastases free survival (DMFS), disease free survival (DFS) and toxicity in early-stage lung cancer patients treated with SBRT in a single tertiary cancer centre.

**Patients and methods:**

We retrospectively evaluated medical records and radiation treatment plan parameters of 228 tumors irradiated in 206 early-stage lung cancer patients between 2016 and 2021 at the Institute of Oncology Ljubljana.

**Results:**

After 25 months of median follow up, 68 of 206 (33%) patients died. Median OS was 46 months (CI 36–56), 1-year, 2-year and 3-year OS were 87%, 74% and 62% and 5-year OS was 31%. A total of 45 disease progressions have been identified in 41 patients. Local progress only was noticed in 5 (2%) patients, systemic progress in 32 (16%) and combined systemic and local in 4 (2%) patients. Local control rate (LCR) at 1 year was 98%, at 2 and 3 years 96% and 95% at 5 years. The 1-, 2- and 3-year LPFS were 98%, 96% and 94%, respectively and 5-year LPFS was 82%. One, 2-, 3- and 5-year DFS were 89%, 81%, 72% and 49%, respectively. Among 28 toxicities recorded only one was Grade 4 (pneumonitis), all others were Grade 1 or 2. No differences in LCR, LPFS, DFS were found in univariate analysis comparing patient, tumor, and treatment characteristics. For OS the only statistically significant difference was found in patients with more than 3 comorbidities compared to those with less comorbidities.

**Conclusions:**

Early lung cancer treated with SBRT at single tertiary cancer centre showed that LCR, LPFS, DFS, DMFS and OS were comparable to published studies. Patients with many comorbidities had significantly worse overall survival compared to those with less comorbidities. No other significant differences by patient, tumor, or treatment characteristics were found for DMFS, LPFS, and DFS. Toxicity data confirmed that treatment was well tolerated.

## Introduction

Localized disease is diagnosed in up to 20% of patients with lung cancer.^1^ This proportion, especially in patients with early lung cancer is increasing due to lung cancer screening programs and covid-19 pandemic's increased lung diagnostics during the last two years. Furthermore, number of inoperable or high-risk patients is growing due to an aging population. According to Slovenian national cancer registry for 2019 localized disease was reported in 18% of all newly diagnosed lung cancer patients.^2^ Standard of care for these patients is lobectomy, however due to comorbidities and old age many of them are not eligible for surgery.^3,4^ Inoperable patients with small tumors and no metastases in local lymph nodes and those who refuse surgery are treated with stereotactic body radiation therapy (SBRT). SBRT is a precise technique that can deliver a very high dose (i.e., ablative dose) to the target volume in one to eight fractions.^5,6^ Studies have shown that efficacy of SBRT can be compared to surgery, although no randomized phase III studies have been completed.^7^ Two prospective studies, STARS and ROSEL, were closed prematurely due to poor accrual.^8^ Combined data with notable limitations from these two trials suggested that SBRT could be a reasonable treatment option in medically operable patients. Recent revised STARS trial with re-accrual of the SABR arm to a larger sample size and follow-up of 5.1 years confirmed the findings.^9^

Additional prospective randomized trials on this topic that will hopefully clarify this issue are STABLE-MATES (sub-lobar resection versus SABR) and VALOR (SABR versus anatomic pulmonary resection), but results will not be ready for some years.^10,11^

The results on the effectiveness of SBRT radiation and standard radiation are contradictory. SPACE trial and LUSTRE trial (published only in abstract form) report no difference in local control and OS.^12,13^ On the other hand, superior local control and OS of SBRT compared to conventional radiotherapy of the primary inoperable peripherally located stage I NSCLC was proved in phase III randomized CHISEL study.^14^

In Slovenia SBRT technique was introduced at the Institute of Oncology Ljubljana in 2016 and its use has been constantly increasing since then. It is used for the treatment of primary tumors, local recurrences and metastases. This report focuses on treatment of early lung cancer patients with SBRT and represents our first 5-year analysis.

## Patients and methods

We retrospectively reviewed medical records of 206 consecutive early-stage lung cancer patients and radiation treatment plan parameters of 228 tumors irradiated with SBRT between 2016 and 2021 at Institute of Oncology Ljubljana. The cut-off date of our analysis was 6^th^ November 2022. In our clinical practice staging investigations routinely included computed tomography (CT) of chest and abdomen, brain CT/magnetic resonance imaging, whole-body fluorodeoxyglucose positron emission tomography (FDG PET/CT), blood work, and pulmonary function tests. All patients had either biopsy-proven lung cancer or pulmonary lesions that were considered “suspicious” by experienced chest radiologist and showed evidence of progression on at least two serial CT imaging studies and/or increased FDG uptake on PET scan. All patients were discussed at the multidisciplinary tumor board. Any decision to proceed with radiation therapy for patients without biopsy confirmation of disease was communicated and agreed upon in multidisciplinary tumor board. Decision was typically based on the predicted probability of malignancy (i.e., enlarged lesion on serial CT scans or PET/CT-avid lesion) and weighed against risks of biopsy. Patients with more than one primary lung tumor without evidence of metastasis were carefully discussed at multidisciplinary tumor board.

### SBRT procedure

All patients undergoing initial 4D-CT simulation (Siemens Somatom Definition AS^®^ CT) required immobilization on T-bar/Wingboard with a vacuum cushion device or thermoplastic mask (for tumors in the apex of the lung). Respiratory motion for tumors in lower lobes was minimized using abdominal compression belt. First two years Novalis Tx linear accelerator (Varian) with Exact Trac verification and correction system was used for detection of patient's movement. After that TrueBeam STx and True beam linear accelerators with the external respiratory monitoring system [Real-time Position Management (RPM) System, Varian^®^ Medical Systems, Palo Alto, CA, USA] and Optical Surface Monitoring System (OSMS) was used. Patients had pre-treatment and verification CBCT image registered to the planning CT for daily position treatment verification. All set-up errors were corrected before treatment delivery.

Internal target volume (ITV) included gross tumor volume (GTV) expended by all visible tumor motion on 4D-CT. The planning target volume (PTV) was generated using a 5 mm circumferential expansion of the ITV. Required covering of ITV was at least 99% of the prescription dose. At least 95% of the PTV volume should be covered with 100% of prescribed dose and at least 99% of PTV volume should be covered with 90% of prescribed dose. Maximum dose was prescribed between 125–150% of the prescribed dose for 1–5 fractions and 110–130% for 8 fractions. The most frequently used energy was 6 MV. During the last two years 6 MV flattening filter free (FFF) became the preferred choice.

Dose restrictions for organ at risk (OAR) were complied according to our inhouse protocol based on AAPM Task Group 101 report, RTOG 0915 study (for 4 fractions), and LungTech study for 8 fractions.^15,16,17^ Restrictions for spinal cord Pmax were taken after Sahgal *et al*.^18^

### Statistical definitions

Local control (LC) was defined as no recurrence within the high-dose region of the primary target tumor volume. LC rate was analyzed for all treated tumors. If a patient had more than one lesion treated, progression of any treated lesions was considered a local recurrence for local progression free survival (LPFS) calculation, which was computed from the date of RT completion till date of local recurrence, last follow up or death. Disease-free survival (DFS) was defined from the date of completed RT treatment to first either systemic or local recurrence of disease, last follow up or death. Distant metastases free survival (DMFS) was calculated from the date of RT completion till date of metastatic spread, last follow up or death. Overall survival (OS) was defined from the date of completed treatment until the date of death or the last contact in months. OS was calculated for each patient regardless of solitary or multiple tumor statuses. The censored cases were defined as the patients still alive at the time of the last follow-up.

The one-, two-, three- and five-year OS, DFS, DMFS and LPFS rates were estimated from the cumulative proportion surviving at the particular time (survival table). All p values ≤ 0.05 were considered statistically significant. Data were analyzed using SPSS 25.0 software (IBM Corp., Armonk, NY, USA).

Radiation-induced toxicity was categorized according to the Common Terminology Criteria for Adverse Events (CTCAE) 5.0.^19^

The study was approved by the Institutional Ethics Committee and Review Board (ERIDNPVO-0025/2022).

## Results

### Patients

Between April 2016 and December 2021, 206 consecutive patients (113 males and 93 females) with 228 tumors were treated at the Institute of Oncology Ljubljana with SBRT due to primary early-stage lung cancer. Mean age of our patients was 71 years (range 53–89). ECOG performance status was mainly good (0–2, 85.4%), although many of them had multiple comorbidities, the vast majority of which were caused by smoking. Most often patients had cardiovascular diseases (ischemic heart disease, heart failure, arterial hypertension, peripheral arterial occlusive disease), lung diseases (chronic obstructive pulmonary disease, interstitial lung disease, emphysema), renal insufficiency and gastrointestinal diseases. Interestingly, almost half of the patients had a concurrent or previous malignancy. We also irradiated a patient with several tumors after a heart transplant.

Patients were inoperable due to impaired lung function (44.7%), comorbidities (41.3%) or both, but 5 patients refused surgical procedure. Lung function data were not available for all patients (Table 1).

**TABLE 1.** Patient characteristics (n = 206)

**Characteristic**	**Number**	**%**
Age mean in years (range)	71.2 (53–89)	
Gender		
Male	113	54.9
Female	93	45.1
Other malignancies^*^		
yes	96	46.6
no	110	53.4
Number of comorbidities		
0–3	125	60.7
> 3	81	39.3
Lung function (%)	mean (range)	
FVC (data available for 153 pts)	87.7 (24–152)	
FEV1 (data available for 164 pts)	61.4 (17–144)	
DLCO (data available for 140 pts)	55.6 (15–120)	
ECOG Performance status before radiotherapy		
0	17	8.3
1	78	37.9
2	81	39.2
3	30	14.6
The reason for non-surgery		
Impaired lung function	92	44.7
Comorbidity	85	41.3
Patient refused	5	2.4
Old age	4	1.9
Combined reasons	20	9.7

* synchronous or in the past

DLCO = diffusing capacity of the lungs for carbon monoxide; ECOG = Eastern Cooperative Oncology Group, FEV1 = forced expiratory volume in 1 second; FVC = forced vital capacity; ptc = patients

### Tumors

Majority of patients (186) had radiation of a single tumor, 18 patients had 2 tumors and 2 patients 3 tumors. Table 2 shows that vast majority of patients (as per AJCC 8th edition) had T1 tumors (85.1%). Adenocarcinomas were present in 84 tumors (36.8%), but nonverified lesions were common as well (36.0%). Tumors were mainly located in upper lobes (66.2%).

**TABLE 2.** Tumor characteristics (n = 228)

**Characteristic**	**Number**	**%**
Histology		
Adenocarcinoma	84	36.8
Squamous cell carcinoma	38	16.7
Small cell lung cancer	4	1.8
NSCLC unspecified	19	8.3
No tissue diagnosis	82	36.0
Carcinoid	1	0.4
Location		
Left upper lobe	78	34.2
Left lower lobe	31	13.6
Right upper lobe	73	32.0
Right middle lobe	11	4.8
Right lower lobe	35	15.4
T stage		
1	194	85.1
2	27	11.8
3	7	3.1

NSCLC = non-small cell lung cancer

### Treatment

Treatment prescription dose was mostly 50–55 Gy in 5 fractions (174 patients), next most common prescription was 54 Gy in 3 fractions (32 patients) and only one patient had a single fraction with 34 Gy delivered. More centrally located tumors were treated with 60 Gy in 8 fractions (16 patients), prescription of 48 Gy in 4 fractions was rarely used (3 patients).

During the first two years only 3D or dynamic conformal arc technique (ARC) was available for SBRT. New linear accelerators made volumetric modulated arc treatment (VMAT) (57.9%) possible afterwards (Table 3).^18^

**TABLE 3.** Treatment characteristics (n = 228)

**Characteristic**		
**Number of fractions**	**Number of tumors**	**%**
1	1	0.4
2	2	0.9
3	32	14.1
4	3	1.3
5	174	76.3
8	16	7.0
**Treatment characteristics**	**Median**	**Mean (range)**
PTV volume (cc)	22.4	30.6 (5.9–160.4)
PTV max dose %	137.5	135.5 (104.7–151.4)
ITV coverage %	100	99.7 (66.5–100)
PTV coverage (V95) %	95	90.4 (32–100)
PTV coverage (V99) %	99.8	99 (88.8–100)
BED	115.5	112.3 (59.5–151.2)
**Technique**	**Number of patents**	**%**
3D	62	27.2
ARC	34	14.9
VMAT	132	57.9

ARC = dynamic conformal arc therapy; BED = biological effective dose; ITV = internal target volume; PTV = planning target volume; VMAT = volumetric modulated arc therapy; 3D = conventional conformal therapy

Median PTV of tumors was 22.4 cubic centimetres (cc) with wide range of size (5.9–160.4). ITV and PTV coverage as well as PTV dose maximum can be found in Table 3.

### Outcomes

Out of 206 patients, 2 patients did not complete intended treatment due to deterioration of medical condition (severe coughing, pleural effusion), but their data were included in the final analysis. They both had only 2 fractions delivered out of 3 fractions planned.

After 25 months of median follow up (range 1–69), 68 of 206 (33%) patients died. Median OS was 46 months (CI 36–56), 1-year, 2-year and 3-year OS were 87%, 74% and 62% and 5-year OS was 31%.

Due to retrospective nature of the analysis cause of death could not be retrieved for 25 patients, others died due to lung cancer (24), covid-19 (6), other malignancies (6), emergencies (4) and COPD (3).

Altogether, 45 recurrences were reported in 41 (20%) patients. Progression was local only in 5 (2%) patients. In two of those local recurrence of simultaneously irradiated 2 tumors was recorded on all irradiated sites at the same time. Systemic progression was noticed in 32 (15%) patients, who had 34 tumors irradiated (2 simultaneously), while in 4 patients (2%) (4 tumors) progressions were combined. Local recurrences (local + combined) showed malignant growth within PTV in 6 tumors and at the edge of PTV within the steep dose gradient in 5 tumors.

**FIGURE 1.**
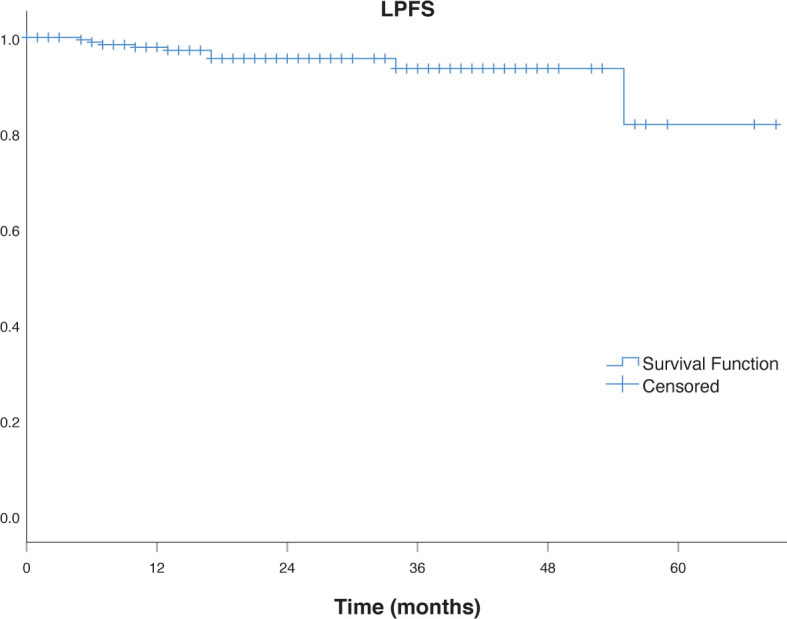
Local progression free survival (LPFS).

Local control rate (LCR) at 1 year was 98%, 96% at 2 and 3 years and 95% at 5 years. Local progression free survival (LPFS) at 1-year, 2-year, 3-year and 5-year were 98%, 96%, 94% and 82%, respectively (Figure 1). Interestingly, among 4 patients with small-cell lung cancer neither had local recurrence or systemic disease during the follow up period.

Among all patients, 36 (18%) experienced systemic disease spread. Distant metastases free survival (DMFS) after 1-year, 2-year and 3-year were 90%, 84% and 74%, while 5-year DMFS was 61%. Systemic spread was noted in lung (28), mediastinal lymph nodes (12), brain (5), bone (5), liver (5), pleura (4), and adrenal gland (3). Eighteen patients with progressive disease were still alive at the cut-off date.

Outcomes showed 89%, 81%, 72% and 49% of DFS after 1-year, 2-years, 3-years and 5-years, respectively.

The following toxicities were reported in 28 patients (13.5%): chest wall pain, rib fracture, dyspnea, pneumonitis, esophagitis, cough, radiodermatitis. Except for one Grade 4 pneumonitis, toxicities were Grade 1 or 2 and of short duration.

**FIGURE 2.**
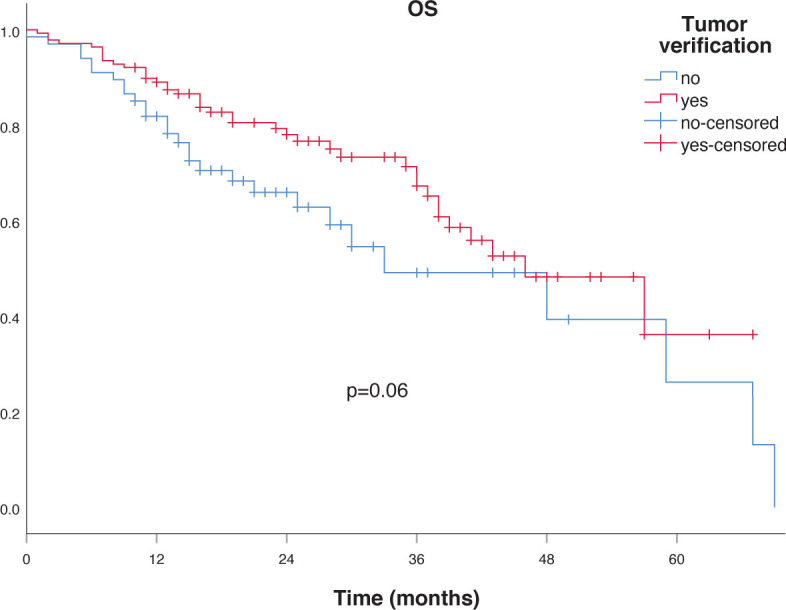
Overall survival (OS) according to tumor verification.

**FIGURE 3.**
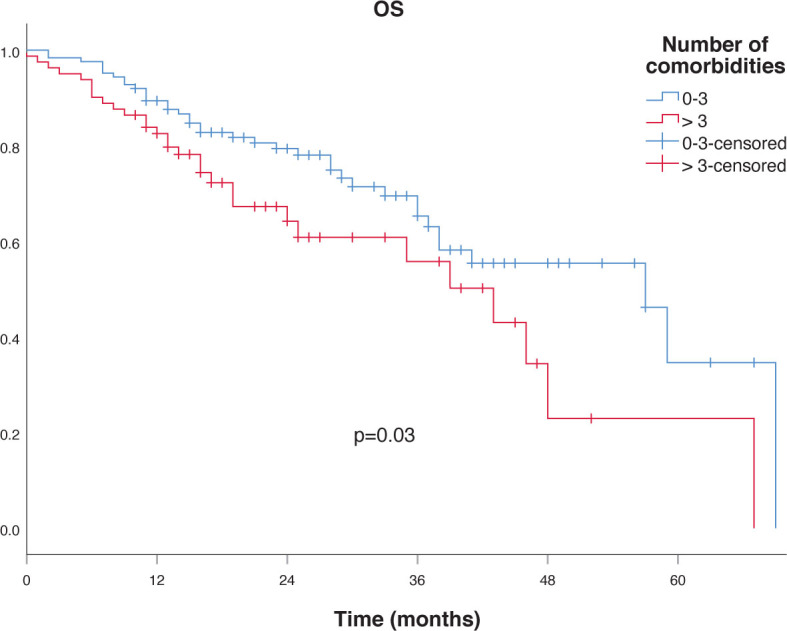
Overall survival (OS) according to number of comorbidities.

To assess the factors affecting LPFS, DFS, DMFS and OS, several clinical and dosimetric factors were studied using univariate analysis, including age, gender, tissue diagnosis, tumor location as well as mean and median PTV size, BED, PTV95 coverage, PTV99 coverage, PTV maximum dose, treatment length and treatment technique. Non-significantly better OS was seen for patients with verified tumors compared to non-verified ones (p = 0.06) as shown in Figure 2 and those with better PS (p = 0.07). The only significant difference in median OS was found between patients with 0–3 comorbidities compared to those with 4 and more, 57 months *vs.* 43 months as shown in Figure 3 (p = 0.03).

## Discussion

SBRT, a noninvasive method of delivering a high ablative radiation dose to a small tumor volume in a few fractions, has become a standard treatment for patients with inoperable early-stage lung cancer over the past two decades.^21,22,23,24^ It also offers a good alternative treatment option for patients who refuse surgery.

Institutions report high local control rates for patients with non-small cell lung cancer, reaching up to 95% in small peripheral tumors and negative nodes after 2–5 years. Our 1-year, 2-year, 3-year and 5-year LCR and LPFS compare favorably with published studies. One of early outcome reports in a single center showed 92% 1-year control rate and 89% at 4-year.^25^ Singh *et al.* reported 1- and 2-year LC rates for all patients to be 92% and 85% respectively.^26^ More recently Abreu *et al.* found 89.1% LC rate after two years.^27^ Latest report from Canadian researchers, who compared 4 different treatment groups (SBRT, hypofractionation, conventional and palliative irradiation) demonstrated that SBRT offered the best local control (94% at 3-years).^28^

Overall survival showed 1-year, 2-year and 3-year after SBRT to be 87%, 74% and 62%, while 5-year OS with 31% was not so favorable, however our cohort of patients included highly comorbid individuals. In already mentioned studies other researchers report 1-year OS of 92%, 2-year 89% and 3-year 67%.^25,27,28^

Resection is the standard treatment for stage I and II lung cancer.^29^ Five-year net survival of patients with localized lung cancer exceeded 60% during the period 2012–2016 in Slovenia.^30^ Introduction of minimally invasive videothoracoscopic surgery represented a revolution in surgical treatment of patients with lung cancer during that period. The latest publication from another surgical center in Slovenia showed that 5-year OS after resection was 70.2% for stage I and 60.2% for stage II.^31^ Our 5-year OS with SBRT is lower, however patients in our analysis were inoperable and with many comorbidities that influenced the outcome.

Patients without treatment have 20% 5-year OS in stage I.^32^ Already ten years ago, Netherland researchers reported 7% decrease in untreated non-small cell lung patients in stage I and 8-month improvement in median survival after introduction of SBRT.^33^ Our OS results in inoperable patients with many comorbidities that would otherwise not be treated show that SBRT will undoubtedly contribute to increased survival rates in stage I and stage II lung cancer in Slovenia in the future.

OS data can be compared to conventional RT. We do not have local data published, but in literature 3D-RT is inferior in terms of OS.^25,28,35^ Moreover, patients with many comorbidities would otherwise only be eligible for palliative radiotherapy or best supportive care. Doupnik *et al.* compared 4 different treatment groups and reported the worst 3-year survival with palliative irradiation (44%), much lower than for SBRT (67%) which is comparable to our 3-year SBRT OS (62%).^28^

We report outcomes with diversified histology of lung lesions, moreover, more than a third of patients had no tissue biopsy. The reason might be that most of our patients were treated during covid-19 epidemic when less pulmonology diagnostics was performed due to the reassignment of pulmonologists to covid wards. While biopsy confirmation remains a goal in the workup of suspected lung tumors and is recommended in all guidelines due to impaired lung function and other comorbidities, in real world situations diagnostic procedure is not possible for up to 25% of patients.^35^ Different histological status of tumors (biopsy proven or not) had no influence on LPFS or DFS in our study. Patients who had verified tumors had better OS compared to non-verified ones, however the difference was not statistically significant (p = 0.06). The reason is probably the patient selection. More patients with poor PS, impaired lung function and other comorbidities had no tumor verification and were also not candidates for treatment after progression, especially systemic treatment. In most of the publications SBRT is presented only for NSCLC data. Retrospective data on histologically unverified early-stage NSCLC lesions treated with SBRT, as opposed to histologically verified ones, showed no significant difference regarding OS and local control while similar rates of DFS and distant failure between pathologically confirmed and presumed NSCLC were observed.^36,37,38^ On the other hand, a large systematic review and meta-analysis of total 43 articles showed lower 3-year overall survival and lower 2-year and 5-year cancer-specific survival for biopsy-proven disease compared to clinical disease. However, 5-year OS was the same for both groups.^39^

The recommended dose and fractionation are determined by tumor volume and location. Median BED delivered to tumors of our patients was 115.5 Gy. In fact, 91.5% of our patients received dose BED (αβ_10_) 100 Gy or higher which has been associated with better outcomes for stage I/II NSCLC.^40,41,42^ Higher dose was not associated with better survival or local tumor control in our analysis.

The optimal duration over which lung SBRT should be delivered is contradictory. Five-fraction SBRT delivered over non-consecutive days showed superior LC and similar toxicity compared to consecutive fractionation in study by Alite *et al*.^43^ On the contrary, Ikawa *et al*. reported beneficial effect on tumor control for consecutive stereotactic body radiotherapy compared to non-consecutive stereotactic body radiotherapy.^44^ No difference in LC was found in group of our patients who completed treatment within one week compared to those whose treatment was longer.

In our analysis, we observed no difference in LPFS or DFS by any patient, tumor, or treatment characteristic. The 5-year OS of 31% was lower than reported in comparable retrospective analyses; however, LPFS was comparable to other outcomes. Again, the reason might be patient selection. Our population of irradiated patients appears to have multiple comorbidities regardless of assessed PS. In fact, significantly better OS was found for patients with less comorbidities. Therefore, in patients with poor PS and significant comorbidities, the benefit of such treatment should carefully be discussed at multidisciplinary tumor board.

### Limitation

Limitations of our study include being retrospective in nature as well as with variation in terms of tumor primary site, size, irradiation dose and histology. No strict imaging evaluation timelines were respected and varied according to clinical scenarios as well as toxicity evaluation. No data about therapy after progression was collected. Patients with more comorbidities had lower OS in our analysis, however no score system was used for calculation and due to retrospective nature of collected data, information might not be accurate.

## Conclusions

Results for LC, LPFS, DFS and OS in our cohort of inoperable early-stage lung cancer patients of different histology treated with SBRT at a single tertiary cancer institution showed comparable results to published studies. Patients with many comorbidities had significantly worse survival compared to those with less comorbidities. No other significant differences by patient, tumor, or treatment characteristics were found for OS, LPFS, and DFS. Toxicity data confirmed that treatment was well tolerated.
